# Similar risk of cardiovascular events in idiopathic inflammatory myopathy and rheumatoid arthritis in the first 5 years after diagnosis

**DOI:** 10.1007/s10067-020-05237-7

**Published:** 2020-06-22

**Authors:** Cristina Párraga Prieto, Fowzia Ibrahim, Richard Campbell, Hector Chinoy, James Galloway, Patrick Gordon

**Affiliations:** 1 Centre for Rheumatic Diseases, King’s College London, London, UK; 2 Rheumatology Department, Salford Royal NHS Foundation Trust, Manchester Academic Health Science Centre, Salford, UK; 3 NIHR Manchester Biomedical Research Centre, Manchester Academic Health Science Centre, Manchester University NHS Foundation Trust, The University of Manchester, Manchester, UK

**Keywords:** Atherosclerosis, Cardiovascular event, Dermatomyositis, Idiopathic inflammatory myopathy, Polymyositis

## Abstract

**Objectives:**

To estimate the incidence of cardiovascular (CV) events in idiopathic inflammatory myopathy (IIM) compared to patients with rheumatoid arthritis (RA) and the general population. To explore the contribution of traditional CV risk factors to any difference observed.

**Methods:**

A retrospective matched population-based cohort study was conducted using UK Clinical Practice Research Datalink (CPRD) from 1987 to 2013. The incidence of CV events was calculated for each cohort over time and compared using Cox proportional hazards models. Multivariable analyses were used to adjust for traditional CV risk factors.

**Results:**

A total of 603 patients with IIM 4047 RA and 4061 healthy controls were included. The rate of CV events in IIM was significantly greater than healthy controls [hazard ratio (HR) 1.47 (95% confidence interval (CI) 1.18–1.83)] and remained significant after adjustment for CV risk factors [HR 1.38 (95% CI 1.11–1.72)]. Risk was similar between IIM and RA [HR 1.01 (95% CI 0.78–1.31)]. The rate of myocardial infarction [HR 1.61 (95% CI 1.27–2.04)] but not stroke [HR 0.92 (95% CI 0.59–1.44)] was significantly greater in IIM compared to healthy controls. After the first 5 years, the rate of CV events for RA remained significantly greater compared to the control group, but appeared to return to that of the healthy controls in the IIM group.

**Conclusion:**

IIM is associated with an increased risk of CV events in the first 5 years after diagnosis similar to that of RA. Beyond 5 years, the risk appears to return to that of the general population in IIM but not RA.

**Table:** 

**Key Points** *• The excess risk of cardiovascular events in IIM is similar to that found in RA.* *• The excess risk of cardiovascular events is greatest in the first 5 years after diagnosis.*

## Introduction

It is well established that several connective tissue diseases and chronic inflammatory disorders are associated with an increased risk for cardiovascular disease (CVD) secondary to accelerated atherosclerosis, including systemic lupus erythematosus (SLE) [1], rheumatoid arthritis (RA) [2] and psoriasis [3]. Traditional risk factors for atherosclerotic vascular disease do not fully explain this increased CVD risk [4–6]. Chronic systemic inflammation is thought to play an important role in accelerating the atherosclerotic processes [7].

However, not all systemic autoimmune diseases carry the same burden. Notably, the risk appears to be greater in SLE compared to RA, likely due to differences in the immune response between these conditions [7].

The idiopathic inflammatory myopathies (IIM) dermatomyositis (DM) and polymyositis (PM) are a group of autoimmune conditions characterised by inflammation of skeletal muscle and frequently other organ systems. DM is distinguished from the other IIMs by its characteristic cutaneous features. The immune response in IIM appears more similar to that of SLE than RA; both are characterised by a relatively normal CRP and may be exacerbated or induced by anti-TNF therapy [8, 9]. In common with SLE and in contrast to RA, a large proportion of IIM patients have raised type 1 interferon levels [10] with a predominant interferon-α gene signature [11]. Data suggest that type 1 interferon may play a role in the premature atherosclerosis seen in SLE [12].

The risk of arterial events and the excess mortality seen in IIM have been compared to that reported in other systemic rheumatic diseases such as RA, ankylosing spondylitis, SLE and systemic sclerosis. However, comparisons are difficult due to the heterogeneity of published articles, and very few population-based reports have been presented [13].

The aims of the study were to estimate incidence of CV events in adult IIM in the UK and compare with RA and general population controls. To assess the differential risk for myocardial infarct (MI) and stroke, as well as magnitude of risk over time, and if an excess risk were established, assess whether the excess risk can be explained by traditional risk factors.

## Materials and methods

### Data source and study design

We conducted a retrospective matched population-based cohort study using the UK Clinical Practice Research Datalink (CPRD) from 1987 to 2013. The CPRD is a governmental, not-for-profit research service, jointly funded by the NHS National Institute for Health Research (NIHR) and the Medicines and Healthcare products Regulatory Agency (MHRA), a part of the Department of Health. CPRD encompasses a large UK dataset with a coverage of 6.9% of UK population and extensively representative of the UK population in terms of age, sex and ethnicity [14]. It has been providing anonymised primary care records for public health research since 1987. In addition, CPRD is linked via a trusted third party (the Health and Social Care Information Centre) to other patient-level datasets from secondary care, disease-specific registry data, socio-economic and mortality records [15]. Hospital Episode Statistics (HES) and UK Death Register were established for the analysis, which respectively provided hospitalisation data and copies of death certificates and ICD-10 codes for primary, secondary and contributory causes of death.

The CPRD has been extensively used for observational research, with numerous studies published in peer-reviewed journals. High validity of CPRD data has been also demonstrated for the study of morbidity, with a median of 89% for confirmed diagnoses [15].

### Patient cohorts

Subjects with at least two consecutive read codes for DM or PM were included as the index cohort (first-ever and subsequent records). As case validation for rarer diseases is an important limitation of the CPRD, detailed validation of IIM was undertaken. Participants were classified as either unlikely, probable or definite for the IIM diagnosis, based upon clinical and laboratory features, performance of muscle biopsy and electromyography, and immunosuppressive therapy, independently by two of the authors (PG and HC). Where disagreement occurred, the classification was agreed following a case discussion. All analyses were limited to probable and definite cases, only. We defined as “probable” those patients with more than one episode of IIM diagnosis recorded on the database (at least two consecutive records of IIM in the clinical or referral record over the study period), high ESR or/and CRP, high CK level, seen in Rheumatology clinic, high dose of steroids treatment and immunosuppressive therapy, and “definite” those with the previous mentioned characteristics in addition to muscle biopsy. Individuals with other autoimmune diseases or conditions that might provide a differential diagnosis for IIM and diagnosis made in those subjects < 18 years old were excluded from all groups. Cases of inclusion body myositis were not included.

Up to four RA patients and healthy subjects on the observational window were matched to each IIM patient (exposed patient) for age (± 5 years) and gender, using the index date matching and based on the initial IIM case records. Cases of IIM who did not fulfil the validation criteria were excluded from all analyses but not their matched RA patient and healthy controls. RA subjects were identified with at least two read codes for the diagnosis, with a “first-ever” and subsequent record of RA in the clinical or referral records during the patient up-to-the date of registration and study period. Clinical and referral records included medical history events and secondary care information, respectively. The index dates of the exposed and RA patients were no more than five years apart. Healthy subjects were defined as those with no records of autoimmune rheumatological disease on CPRD, using the validated CPRD coding system. Participants of this group were set to be the index date of the exposed patient.

### Assessment of cardiovascular events

The outcomes of interest were fatal and non-fatal first incident major cardiovascular events during the period of follow-up, including myocardial infarction, acute coronary syndrome (ACS), unstable angina or stroke (ischaemic and haemorrhagic cerebrovascular events). Medical codes of CPRD, ICD-9 410 and ICD-10 121 codes were used to identify the mentioned CV events. Patients with CV events and/or stroke prior to the diagnosis of the autoimmune disease (IIM or RA) were excluded for both analyses (CV event and stroke analysis). In our main analysis, the combined cardiovascular endpoint was identified using read codes in CPRD as well as linked data from the Office for National Statistics death register and also hospitalisation data from Hospital Episode Statistics. Secondary analyses considered outcomes for cardiac and stroke endpoints separately.

Comorbidity was based on medical records, coded using read codes of CPRD as per previous published studies [16]. Obesity was defined by the recorded diagnosis and/or body mass index on the CPRD database according to WHO categories. Concerning tobacco consumption, patients were classified as active smoker, ex-smoker and never-smoker, consistent with the CPRD classification criteria. With regard to steroids treatment, we included those patients who had ever been on oral or systemic glucocorticosteroids over the study period.

### Statistical analysis

Kruskal–Wallis and Chi-square tests were used to assess significant differences in demographic variables and comorbid medical disorders between patients with IIM, RA and healthy controls, using the former test to compare those continuous variables and the latter for categorical variables between groups. The incidence of cardiovascular events was calculated for each cohort over time and compared using Cox proportional hazards models. The validity of the Cox models were tested graphically using Nelson Aalen plots. Multivariable analyses were used to adjust for traditional cardiac risk factors (age, gender, diabetes mellitus, hypertension and smoking). Multivariable analyses adjusted for a priori chosen confounders as follows: age, gender, smoking status, prior cardiovascular disease (including myocardial infarction, acute coronary syndrome, unstable angina or stroke) and diabetes. Acknowledging that statin use may lie on the causal pathway to IIM, adjustment for statins or hypercholesterolaemia was not performed. However, we did not consider removing all patients on statin treatment from the analysis due to the possibility of causing a selection bias. In addition, statin-induced myopathy is a small subpopulation of myositis and so unlikely to have a major impact.

## Results

The initial search of the CPRD revealed 1013 subjects with at least two read codes for IIM. Review of these records excluded 199 as the diagnosis was deemed neither probable nor definite. A further 211 subjects were excluded due to other diagnoses (e.g. systemic sclerosis or polymyalgia rheumatica). In total, 603 subjects with IIM remained eligible for inclusion in the main analysis, 300 patients with DM (50%) and 303 with PM (50%). In addition, 4047 patients with RA and 4061 healthy controls were included. The baseline characteristics of these groups are shown in Table 1.

**Table 1:** Baseline characteristics

Characteristic	Controls *n* = 4061	IIM patients *n* = 603	*P* value*	RA patients *n* = 4047	*P* value**
Age (years)	52 ± 16	58 ± 16	0.0001	57 ± 15	0.0001
Female	2567 (63)	386 (64)	0.703	2597 (64)	0.369
Current smoker	1019 (25)	154 (26)	0.024	1446 (36)	< 0.001
Hypertension	1352 (33)	253 (42)	< 0.001	1506 (37)	< 0.001
Diabetes	600 (15)	132 (22)	< 0.001	796 (20)	< 0.001
Statin therapy	87 (2)	109 (18)	< 0.001	511 (13)	< 0.001
Obese	317 (8)	70 (12)	0.002	415 (10)	< 0.001
GC at baseline	476 (12)	433 (67)	< 0.001	2095 (52)	< 0.001

Values are expressed as the mean ± SD for age and *n* (%) for the rest of characteristics

*IIM*, idiopathic inflammatory myopathy; RA, rheumatoid arthritis; GC, Glucocorticoids at baseline

*IIM compared to control group; **RA compared to control group

Patients diagnosed with IIM and RA were significantly older than controls within the expected range of difference (± 5 years) and had significantly more traditional risk factors for cardiovascular disease (Table 1). There were significantly more current smokers (*p* < 0.0001) in the RA compared to the IIM group. There was more statin use (*p* < 0.0001) and hypertension (*p* = 0.025) in the IIM group compared to the RA group but otherwise no significant differences observed for the variables between these two groups. Of the healthy individuals, 12% had used glucocorticoids (Table 1).

The rate of cardiovascular events (Table 2) was higher in the IIM and RA cohort (2.9 and 2.7 per 100 patient years, respectively) compared to the control group (1.5 per 100 patient years). IIM and RA patients had significantly higher hazard ratio (HRs) for all cardiovascular events: age and gender adjusted [HR 1.47 (95% CI 1.18–1.83)] for IIM and [HR 1.36 (95% CI 1.22–1.52)] for RA. As expected, adjustment for baseline cardiovascular risk factors reduced both HRs but there remained a significant increased risk compared to the control group. An analysis limited to cardiac events showed similar results with significantly higher HRs for cardiac events for both IIM and RA, before and after adjustment for cardiovascular risk factors (Table 2). Analysis of stroke revealed no significant increased risk in IIM or RA patients compared to controls.

**Table 2:** Cardiovascular risk in myositis cohort compared to rheumatoid arthritis and healthy controls

Outcome	IIM cohort	RA cohort	Controls
Any CV^1^ Event			
Number of patients	94	700	652
Exposure (years)	3244	25,785	42,871
Incidence^2^ (95%CI)	2.90 (2.37–3.54)	2.71 (2.52–2.92)	1.52 (1.41–1.64)
Unadjusted HR (95%CI)	1.47 (1.18–1.83)	1.36 (1.22–1.52)	Ref
Adjusted HR^3^ (95%CI)	1.38 (1.11–1.72)	1.26 (1.12–1.41)	Ref
Myocardial infarction			
Number of patients	82	559	482
Exposure (years)	3287	26,545	43,823
Incidence^2^ (95%CI)	2.49 (2.01–3.10)	2.11 (1.94–2.30)	1.10 (1.01–1.20)
Unadjusted HR (95%CI)	1.73 (1.37–2.20)	1.47 (1.29–1.67)	Ref
Adjusted HR^3^ (95%CI)	1.61 (1.27–2.04)	1.36 (1.19–1.54)	Ref
Stroke			
Number of patients	21	209	241
Exposure (years)	3554	28,323	46,114
Incidence^2^ (95%CI)	0.59 (0.39–0.91)	0.73 (0.64–0.84)	0.52 (0.46–0.59)
Unadjusted HR (95%CI)	0.92 (0.59–1.44)	1.11 (0.91–1.34)	Ref
Adjusted HR^3^ (95%CI)	0.88 (0.56–1.39)	1.06 (0.87–1.29)	Ref

*IIM*, idiopathic inflammatory myopathy; RA, rheumatoid arthritis

^1^Cardiovascular event (CV); ^2^Incidence per 100 person-time; ^3^Adjusted hazard ratio (HR) for age, gender, diabetes, hypertension, smoking status

The adjusted overall risk for a cardiovascular event showed similar trends in IIM and RA groups [HR 1.38 (95% CI 1.11–1.72) for IIM and HR 1.26 (95% CI 1.12–1.41) for RA], with a non-significant difference between the two diseases.

**Fig. 1:**
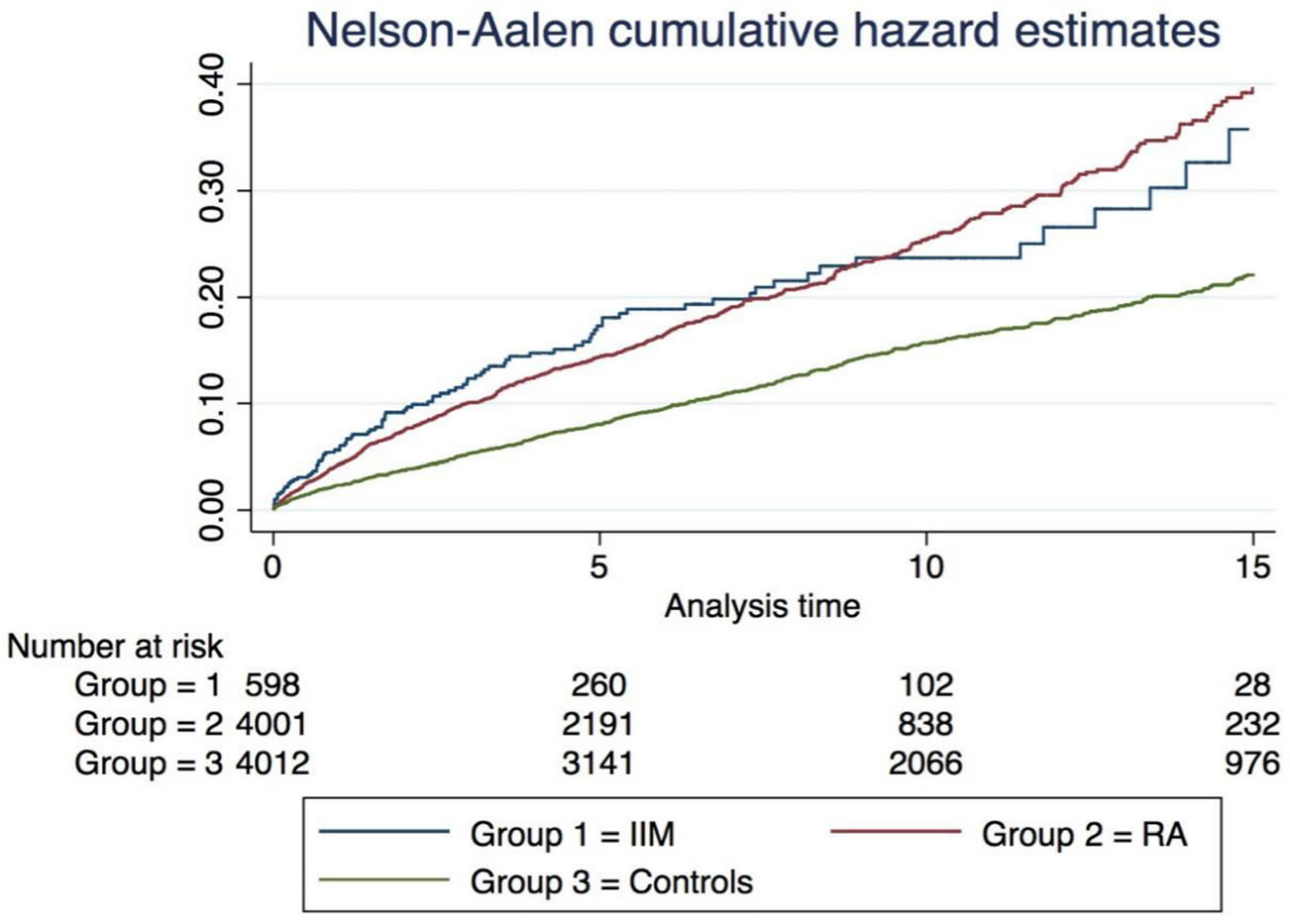
Cardiovascular events over time

An analysis of cardiovascular event rates over time is shown in Fig. 1. The median follow-up per person was 7 years. The hazard for cardiovascular events increased in all groups over time. The excess hazard in IIM was most apparent in the first 5 years, [adjusted HR 1.32 (95% CI 0.90–1.95)], but then appeared to diminish in the 5–10 year period [0.74 (0.32–1.69)] and 10–15 year period [1.03 (0.57–1.84)]. In contrast, in RA patients, the difference in hazard was more stable over time: first 5 years [adjusted HR 1.22 (95% CI 0.91–1.65)], 5–10 years [1.22 (0.92–1.63)] and 10–15 years [1.31 (1.05–1.65)] (Table 3).

**Table 3:** Cardiovascular event rates over time

Time window	IIM	RA	Controls
First 5 years			
Number of patients	74	464	292
Exposure (years)	1972	15,407	15,407
Incidence^1^ (95%CI)	3.75 (2.99–4.71)	3.01 (2.75–3.30)	1.63 (1.45–1.82)
Unadjusted HR (95%CI)	1.72 (1.33–2.22)	1.34 (1.18–1.59)	Ref
Adjusted HR^2^ (95%CI)	1.32 (0.90–1.95)	1.22 (0.91–1.65)	Ref
Second 5 years			
Number of patients	6	97	94
Exposure (years)	504	4119	6548
Incidence^1^ (95%CI)	1.19 (0.53–2.65)	2.35 (1.93–2.87)	1.43 (1.17–1.76)
Unadjusted HR (95%CI)	0.77 (0.34–1.75)	1.39 (1.05–1.85)	Ref
Adjusted HR^2^ (95%CI)	0.74 (0.32–1.69)	1.22 (0.92–1.63)	Ref
Third 5 years			
Number of patients	12	128	199
Exposure (years)	682	5555	14,015
Incidence^1^ (95%CI)	1.76 (1.00–3.10)	2.30 (1.94–2.74)	1.42 (1.24–1.63)
Unadjusted HR (95%CI)	1.08 (0.60–1.94)	1.43 (1.14–1.79)	Ref
Adjusted HR^2^ (95%CI)	1.03 (0.57–1.84)	1.31 (1.05–1.65)	Ref

IIM, idiopathic inflammatory myopathy; *RA,* rheumatoid arthritis

^1^Incidence per 100 person-time; ^2^Adjusted hazard ratio (HR) for age, gender, diabetes, hypertension, smoking status

## Discussion

In this large and retrospective population-based study, IIM was associated with an increased risk of developing a cardiovascular event. Of note, the increased HR in the IIM cohort remained significant in multivariate analyses, suggesting that the excess risk early on cannot be explained entirely by traditional CV risk factors and suggests a role for inflammation in the increased CVD risk in IIM.

Previous studies have suggested an increased incidence of cardiovascular disease in myositis. In a study from Taiwan, 907 DM patients diagnosed from a national insurance claims database had an adjusted HR 3.37 for MI compared tocontrols in the first 2 years [17]. However, this study did not exclude patients with other connective tissue diseases or validate the diagnosis beyond the ICD code; perhaps for these reasons, the incidence of DM was over twice that described for all IIM in another Taiwanese study [18]. A Swedish study looked at patients hospitalised for autoimmune disease in Sweden; the standardised incident ratio for subsequent hospitalisation for coronary heart disease was 3.81 in the first year, also higher than our estimates. Nevertheless, again this study did not exclude patients with other connective tissue diseases or validate the diagnosis beyond the ICD code. In addition, the study assessed patients with severe disease requiring hospital admission [19]. A further study of 607 Canadian IIM patients, which used billing, hospitalisation and pharmacy databases, had slightly more stringent inclusion criteria and had an age- and sex-adjusted standardised incident ratio compared to the normal population of 1.95, more in keeping with our findings [20]. A study from British Columbia which used ICD codes alone to validate cases, identified 774 new cases of IIM over 14 years in a population of 4.7 million, over twice the expected figure [21]. They reported a HR for MI of 3.89 for PM, 2.92 for DM and non-increased risk for ischaemic stroke in IIM. This study showed similar, but not significant trends between the increased risk for MI and stroke in DM and PM patients compared to the general population. A smaller number of outcomes was reported as the possible reason for their results. Another hypothesis of the discrepancy between our results and other studies is the inclusion of ischaemic and haemorrhagic stroke in our analysis instead of just ischaemic cerebrovascular events [17, 22, 23]. The limitation of stroke event ascertainment may have also contributed to our findings.

In previous large meta-analyses, rheumatoid arthritis has been associated with a 48% increased risk of cardiovascular events (relative risk 1.48, 95% CI 1.36–1.62) compared with the general population [24]. We compared the incidence of CV events in IIM with controls and RA, enabling comparison of the incidence with more a studied disease, providing an anchor point for the data and internal validation. Although the adjusted overall risk for a cardiovascular event was slightly greater for IIM than for RA in our study, the difference was small and not significant. As IIM may occur in association with other connective tissue diseases including SLE and systemic sclerosis, we excluded patients with additional diagnoses of connective tissue disease. Due to the data available on the CPRD, we were also able to be more stringent in our case assignment. As a result of this approach, we identified fewer than expected cases which may have led to an unexpected case selection bias and reduced the power of the study. This might have contributed to the difference between the excess of increased CV risk seen in IIM patients in comparison with RA cohort in our research. However, it also highlights the complexity of autoimmune diseases in their pathogenesis, with common factors in their aetiology but different mechanisms of autoimmunity.

Notably, whilst the HR for a cardiovascular event in RA was fairly stable across the 15-year period after diagnosis, the risk for IIM varied markedly over time. The HR was increased in IIM compared to RA and healthy controls in the first 5 years but subsequently appeared to return to that of the general population. Similar results have been suggested in prior IIM studies, in particular, beyond the first year follow-up [25]. Rai et al. also reported a significant attenuation of the cardiovascular risk in the 5 years following the PM diagnosis, not seen in the DM group [21]. These results would be more in keeping with the observed perivascular changes, endothelial damage and myocardial involvement of IIM rather than the traditional pattern of premature atherosclerosis.

Disease activity is a predictor of cardiovascular disease in many inflammatory diseases including RA and SLE [26–28]. If the same is true for IIM, one might hypothesise that disease activity is greatest in the first 5 years subsequently settling with the CV risk following suit. Nevertheless, whilst the disease is perhaps likely to be at its most inflammatory in the initial stages, the limited literature available to date does not suggest a resolution of the inflammation after 5 years. Over a 20-year period, Sultan and colleagues reported that 67.5% of the surviving patients in their cohort had a chronic relapsing-remitting course or chronic progressive disease, with only 17.5% going into complete remission [29]. In juvenile dermatomyositis (JDM), Sanner and colleagues reported that after 16.8 years, 51–73% of JDM patients still had active disease [30]. Whilst steroid use is almost universal in the initial management of both rheumatoid and IIM, the initial dosage is generally much higher in IIM and is a potential cause for the higher initial CVD risk in IIM patients.

A further possibility is that in the early stages of IIM, raised troponin levels either due to myocarditis or a high creatine kinase may lead to the incorrect diagnosis of a non-ST elevation myocardial infarct [31, 32]. This might lead to false high-recorded MI event rates. In keeping with this hypothesis, a cardiac MR study showed 62% of an IIM cohort had inflammatory cardiac lesions [33]. However, the results need to be interpreted in the context of the CPRD data limitations.

There are a number of limitations associated with our study. Case ascertainment bias and also survivorship bias, which may impact on event rates over time, cannot be excluded as partial explanations for the findings observed. In addition, there was significant attrition in numbers over the later periods (from 5 to 10 and 10 to 15 years) which affected the power of our study in these periods of time.

Our database is UK only and therefore might be subject to bias relating to the UK population, including local prescribing and treatment practices for this condition which could impact on cardiac risk. Notably, a high proportion of the healthy individuals (12%) included in our study had used glucocorticoids. In comparison, a previous CPRD study of the use of oral corticosteroids in the UK [34] reported a prevalence of oral corticosteroid treatment in the total adult CPRD population of 0.9%, increasing up to 2.5% in the elderly population. In the vast majority of the cases, the indication of that therapy was due to respiratory disease, being prescribed by the General Practitioners of which prescriptions are automatically collected on the CPRD. As such, these results may not be generalisable to other IIM populations.

Further studies, ideally in large well-characterised prospective cohort, are required to confirm the reduction of the cardiovascular risk following 5 years the initial IIM diagnosis and to ensure that the events early in the disease are atherosclerotic rather than inflammatory.

In conclusion, our study demonstrated an excess of cardiovascular events in IIM of a similar order to that found in RA, with a greater excess risk during the first 5 years following the diagnosis. Consequently, guidelines on the assessment and management in IIM need to be developed to take into account cardiovascular risk in IIM. This would help reduce mortality in IIM which remains high [35, 36] despite immunosuppressive treatment, with cardiac disease being a major cause of death [37–39].
